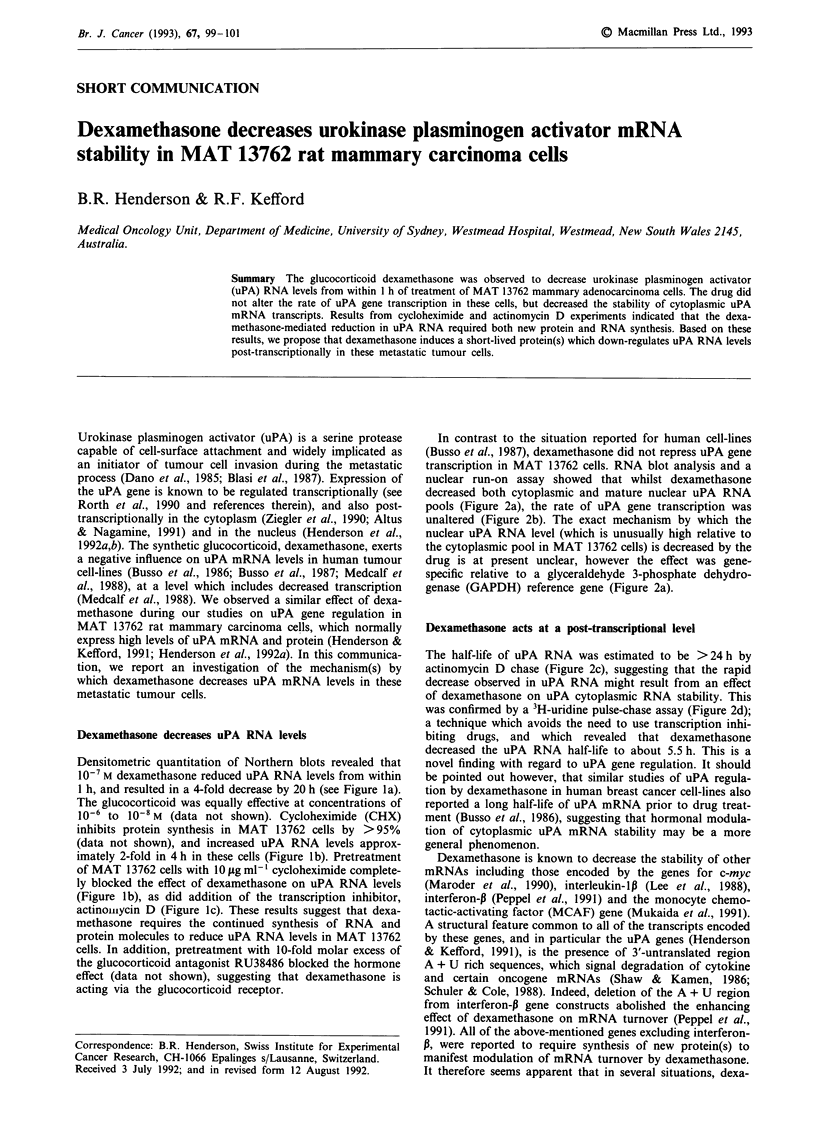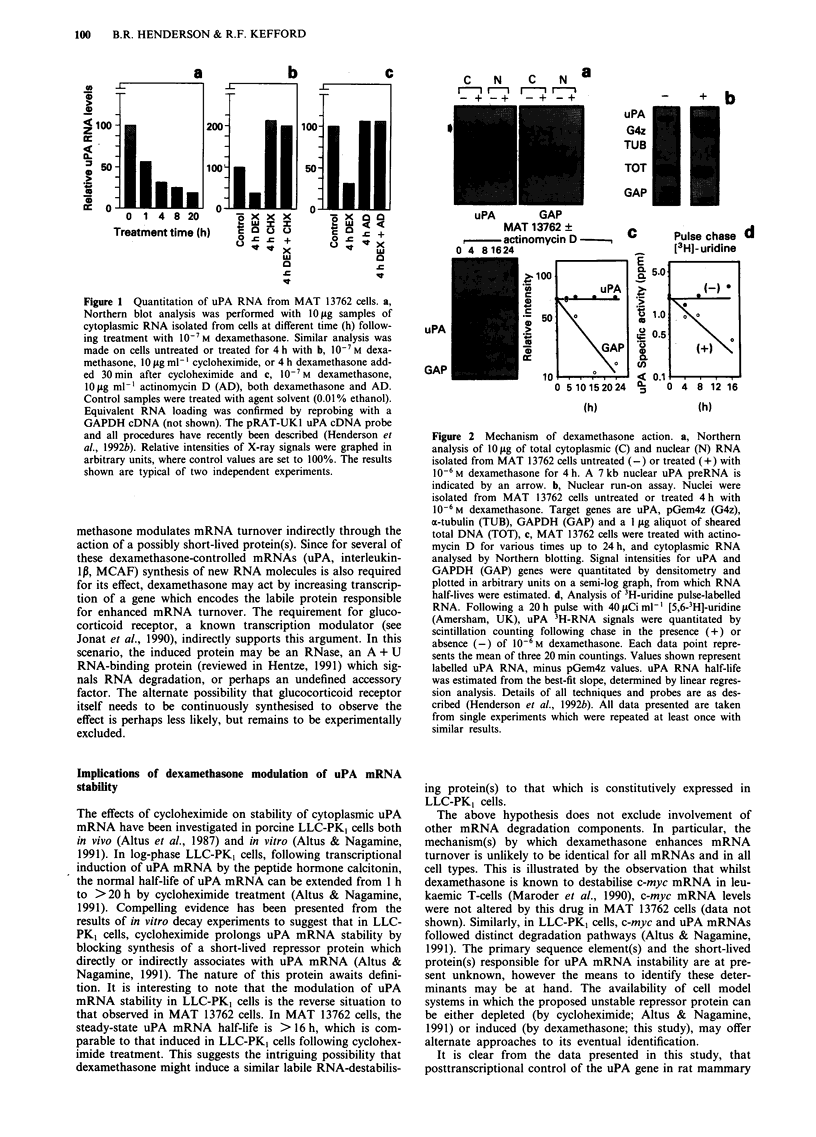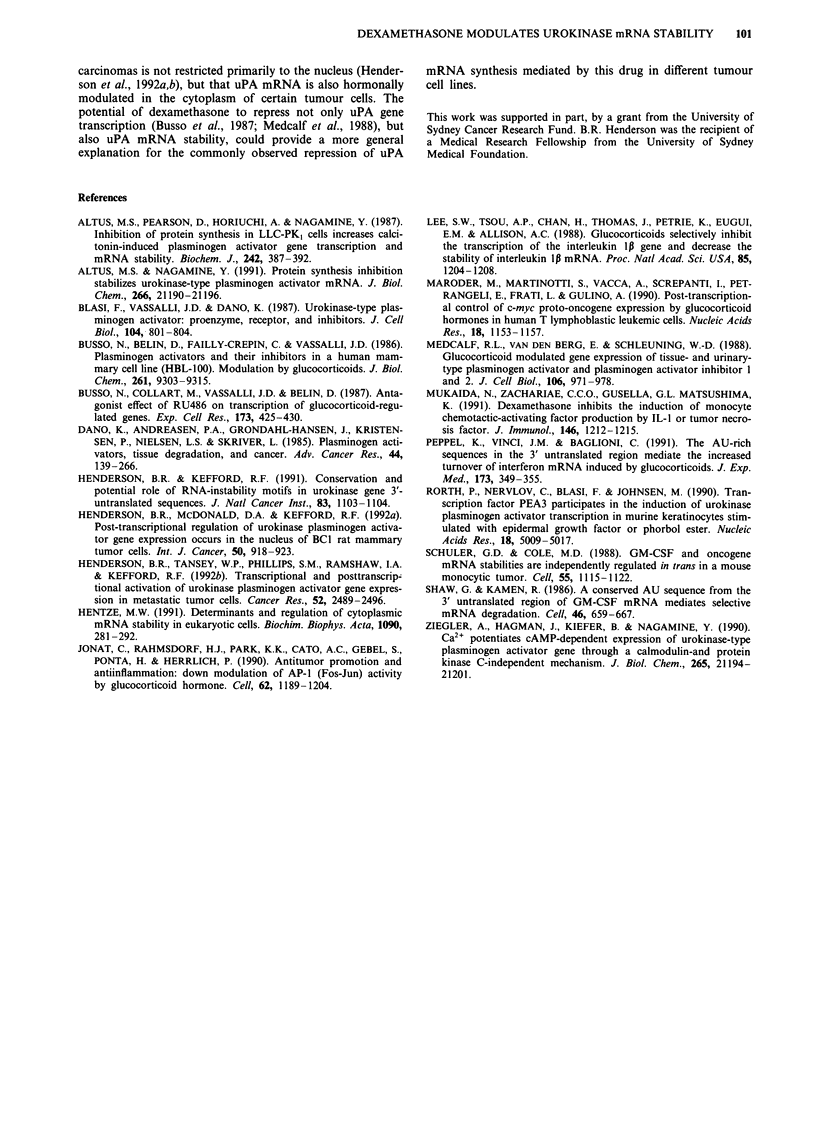# Dexamethasone decreases urokinase plasminogen activator mRNA stability in MAT 13762 rat mammary carcinoma cells.

**DOI:** 10.1038/bjc.1993.16

**Published:** 1993-01

**Authors:** B. R. Henderson, R. F. Kefford

**Affiliations:** Department of Medicine, University of Sydney, Westmead Hospital, New South Wales, Australia.

## Abstract

**Images:**


SHORT COMMUNICATION

### Dexamethasone decreases urokinase plasminogen activator mRNA stability in MAT 13762 rat mammary carcinoma cells

B.R. Henderson & R.F. Kefford

*Medical Oncology Unit, Department of Medicine, University of Sydney, Westmead Hospital, Westmead, New South Wales 2145, Australia.*

**Summary** The glucocorticoid dexamethasone was observed to decrease urokinase plasminogen activator (uPA) RNA levels from within 1 h of treatment of MAT 13762 mammary adenocarcinoma cells. The drug did not alter the rate of uPA gene transcription in these cells, but decreased the stability of cytoplasmic uPA mRNA transcripts. Results from cycloheximide and actinomycin D experiments indicated that the dexamethasone-mediated reduction in uPA RNA required both new protein and RNA synthesis. Based on these results, we propose that dexamethasone induces a short-lived protein(s) which down-regulates uPA RNA levels post-transcriptionally in these metastatic tumour cells.

Urokinase plasminogen activator (uPA) is a serine protease capable of cell-surface attachment and widely implicated as an initiator of tumour cell invasion during the metastatic process (Dano *et al.*, 1985; Blasi *et al.*, 1987). Expression of the uPA gene is known to be regulated transcriptionally (see Rorth *et al.*, 1990 and references therein), and also post-transcriptionally in the cytoplasm (Ziegler *et al.*, 1990; Altus & Nagamine, 1991) and in the nucleus (Henderson *et al.*, 1992*a*,*b*). The synthetic glucocorticoid, dexamethasone, exerts a negative influence on uPA mRNA levels in human tumour cell-lines (Busso *et al.*, 1986; Busso *et al.*, 1987; Medcalf *et al.*, 1988), at a level which includes decreased transcription (Medcalf *et al.*, 1988). We observed a similar effect of dexamethasone during our studies on uPA gene regulation in MAT 13762 rat mammary carcinoma cells, which normally express high levels of uPA mRNA and protein (Henderson & Kefford, 1991; Henderson *et al.*, 1992*a*). In this communication, we report an investigation of the mechanism(s) by which dexamethasone decreases uPA mRNA levels in these metastatic tumour cells.

### Dexamethasone decreases uPA RNA levels

Densitometric quantitation of Northern blots revealed that $10^{-7}$ M dexamethasone reduced uPA RNA levels from within 1 h, and resulted in a 4-fold decrease by 20 h (see Figure 1a). The glucocorticoid was equally effective at concentrations of $10^{-6}$ to $10^{-8}$ M (data not shown). Cycloheximide (CHX) inhibits protein synthesis in MAT 13762 cells by >95% (data not shown), and increased uPA RNA levels approximately 2-fold in 4 h in these cells (Figure 1b). Pretreatment of MAT 13762 cells with 10 $\mu$g ml$^{-1}$ cycloheximide completely blocked the effect of dexamethasone on uPA RNA levels (Figure 1b), as did addition of the transcription inhibitor, actinomycin D (Figure 1c). These results suggest that dexamethasone requires the continued synthesis of RNA and protein molecules to reduce uPA RNA levels in MAT 13762 cells. In addition, pretreatment with 10-fold molar excess of the glucocorticoid antagonist RU38486 blocked the hormone effect (data not shown), suggesting that dexamethasone is acting via the glucocorticoid receptor.

Correspondence: B.R. Henderson, Swiss Institute for Experimental Cancer Research, CH-1066 Epalinges s/Lausanne, Switzerland.
Received 3 July 1992; and in revised form 12 August 1992.

In contrast to the situation reported for human cell-lines (Busso *et al.*, 1987), dexamethasone did not repress uPA gene transcription in MAT 13762 cells. RNA blot analysis and a nuclear run-on assay showed that whilst dexamethasone decreased both cytoplasmic and mature nuclear uPA RNA pools (Figure 2a), the rate of uPA gene transcription was unaltered (Figure 2b). The exact mechanism by which the nuclear uPA RNA level (which is unusually high relative to the cytoplasmic pool in MAT 13762 cells) is decreased by the drug is at present unclear, however the effect was gene-specific relative to a glyceraldehyde 3-phosphate dehydrogenase (GAPDH) reference gene (Figure 2a).

### Dexamethasone acts at a post-transcriptional level

The half-life of uPA RNA was estimated to be >24 h by actinomycin D chase (Figure 2c), suggesting that the rapid decrease observed in uPA RNA might result from an effect of dexamethasone on uPA cytoplasmic RNA stability. This was confirmed by a $^3$H-uridine pulse-chase assay (Figure 2d); a technique which avoids the need to use transcription inhibiting drugs, and which revealed that dexamethasone decreased the uPA RNA half-life to about 5.5 h. This is a novel finding with regard to uPA gene regulation. It should be pointed out however, that similar studies of uPA regulation by dexamethasone in human breast cancer cell-lines also reported a long half-life of uPA mRNA prior to drug treatment (Busso *et al.*, 1986), suggesting that hormonal modulation of cytoplasmic uPA mRNA stability may be a more general phenomenon.

Dexamethasone is known to decrease the stability of other mRNAs including those encoded by the genes for c-*myc* (Maroder *et al.*, 1990), interleukin-1$\beta$ (Lee *et al.*, 1988), interferon-$\beta$ (Peppel *et al.*, 1991) and the monocyte chemotactic-activating factor (MCAF) gene (Mukaida *et al.*, 1991). A structural feature common to all of the transcripts encoded by these genes, and in particular the uPA genes (Henderson & Kefford, 1991), is the presence of 3′-untranslated region A + U rich sequences, which signal degradation of cytokine and certain oncogene mRNAs (Shaw & Kamen, 1986; Schuler & Cole, 1988). Indeed, deletion of the A + U region from interferon-$\beta$ gene constructs abolished the enhancing effect of dexamethasone on mRNA turnover (Peppel *et al.*, 1991). All of the above-mentioned genes excluding interferon-$\beta$, were reported to require synthesis of new protein(s) to manifest modulation of mRNA turnover by dexamethasone. It therefore seems apparent that in several situations, dexa-

**Figure 1** Quantitation of uPA RNA from MAT 13762 cells. **a**, Northern blot analysis was performed with 10 µg samples of cytoplasmic RNA isolated from cells at different time (h) following treatment with $10^{-7}$ M dexamethasone. Similar analysis was made on cells untreated or treated for 4 h with **b**, $10^{-7}$ M dexamethasone, 10 µg $ml^{-1}$ cycloheximide, or 4 h dexamethasone added 30 min after cycloheximide and **c**, $10^{-7}$ M dexamethasone, 10 µg $ml^{-1}$ actinomycin D (AD), both dexamethasone and AD. Control samples were treated with agent solvent (0.01% ethanol). Equivalent RNA loading was confirmed by reprobing with a GAPDH cDNA (not shown). The pRAT-UK1 uPA cDNA probe and all procedures have recently been described (Henderson *et al.*, 1992*b*). Relative intensities of X-ray signals were graphed in arbitrary units, where control values are set to 100%. The results shown are typical of two independent experiments.

**Figure 2** Mechanism of dexamethasone action. **a**, Northern analysis of 10 µg of total cytoplasmic (C) and nuclear (N) RNA isolated from MAT 13762 cells untreated (−) or treated (+) with $10^{-6}$ M dexamethasone for 4 h. A 7 kb nuclear uPA preRNA is indicated by an arrow. **b**, Nuclear run-on assay. Nuclei were isolated from MAT 13762 cells untreated or treated 4 h with $10^{-6}$ M dexamethasone. Target genes are uPA, pGem4z (G4z), α-tubulin (TUB), GAPDH (GAP) and a 1 µg aliquot of sheared total DNA (TOT), **c**, MAT 13762 cells were treated with actinomycin D for various times up to 24 h, and cytoplasmic RNA analysed by Northern blotting. Signal intensities for uPA and GAPDH (GAP) genes were quantitated by densitometry and plotted in arbitrary units on a semi-log graph, from which RNA half-lives were estimated. **d**, Analysis of $^3H$-uridine pulse-labelled RNA. Following a 20 h pulse with 40 µCi $ml^{-1}$ [5,6-$^3H$]-uridine (Amersham, UK), uPA $^3H$-RNA signals were quantitated by scintillation counting following chase in the presence (+) or absence (−) of $10^{-6}$ M dexamethasone. Each data point represents the mean of three 20 min countings. Values shown represent labelled uPA RNA, minus pGem4z values. uPA RNA half-life was estimated from the best-fit slope, determined by linear regression analysis. Details of all techniques and probes are as described (Henderson *et al.*, 1992*b*). All data presented are taken from single experiments which were repeated at least once with similar results.

methasone modulates mRNA turnover indirectly through the action of a possibly short-lived protein(s). Since for several of these dexamethasone-controlled mRNAs (uPA, interleukin-1β, MCAF) synthesis of new RNA molecules is also required for its effect, dexamethasone may act by increasing transcription of a gene which encodes the labile protein responsible for enhanced mRNA turnover. The requirement for glucocorticoid receptor, a known transcription modulator (see Jonat *et al.*, 1990), indirectly supports this argument. In this scenario, the induced protein may be an RNase, an A + U RNA-binding protein (reviewed in Hentze, 1991) which signals RNA degradation, or perhaps an undefined accessory factor. The alternate possibility that glucocorticoid receptor itself needs to be continuously synthesised to observe the effect is perhaps less likely, but remains to be experimentally excluded.

**Implications of dexamethasone modulation of uPA mRNA stability**

The effects of cycloheximide on stability of cytoplasmic uPA mRNA have been investigated in porcine LLC-$PK_1$ cells both *in vivo* (Altus *et al.*, 1987) and *in vitro* (Altus & Nagamine, 1991). In log-phase LLC-$PK_1$ cells, following transcriptional induction of uPA mRNA by the peptide hormone calcitonin, the normal half-life of uPA mRNA can be extended from 1 h to >20 h by cycloheximide treatment (Altus & Nagamine, 1991). Compelling evidence has been presented from the results of *in vitro* decay experiments to suggest that in LLC-$PK_1$ cells, cycloheximide prolongs uPA mRNA stability by blocking synthesis of a short-lived repressor protein which directly or indirectly associates with uPA mRNA (Altus & Nagamine, 1991). The nature of this protein awaits definition. It is interesting to note that the modulation of uPA mRNA stability in LLC-$PK_1$ cells is the reverse situation to that observed in MAT 13762 cells. In MAT 13762 cells, the steady-state uPA mRNA half-life is >16 h, which is comparable to that induced in LLC-$PK_1$ cells following cycloheximide treatment. This suggests the intriguing possibility that dexamethasone might induce a similar labile RNA-destabilising protein(s) to that which is constitutively expressed in LLC-$PK_1$ cells.

The above hypothesis does not exclude involvement of other mRNA degradation components. In particular, the mechanism(s) by which dexamethasone enhances mRNA turnover is unlikely to be identical for all mRNAs and in all cell types. This is illustrated by the observation that whilst dexamethasone is known to destabilise c-*myc* mRNA in leukaemic T-cells (Maroder *et al.*, 1990), c-*myc* mRNA levels were not altered by this drug in MAT 13762 cells (data not shown). Similarly, in LLC-$PK_1$ cells, c-*myc* and uPA mRNAs followed distinct degradation pathways (Altus & Nagamine, 1991). The primary sequence element(s) and the short-lived protein(s) responsible for uPA mRNA instability are at present unknown, however the means to identify these determinants may be at hand. The availability of cell model systems in which the proposed unstable repressor protein can be either depleted (by cycloheximide; Altus & Nagamine, 1991) or induced (by dexamethasone; this study), may offer alternate approaches to its eventual identification.

It is clear from the data presented in this study, that posttranscriptional control of the uPA gene in rat mammary carcinomas is not restricted primarily to the nucleus (Henderson *et al.*, 1992*a,b*), but that uPA mRNA is also hormonally modulated in the cytoplasm of certain tumour cells. The potential of dexamethasone to repress not only uPA gene transcription (Busso *et al.*, 1987; Medcalf *et al.*, 1988), but also uPA mRNA stability, could provide a more general explanation for the commonly observed repression of uPA mRNA synthesis mediated by this drug in different tumour cell lines.

This work was supported in part, by a grant from the University of Sydney Cancer Research Fund. B.R. Henderson was the recipient of a Medical Research Fellowship from the University of Sydney Medical Foundation.

### References

ALTUS, M.S., PEARSON, D., HORIUCHI, A. & NAGAMINE, Y. (1987). Inhibition of protein synthesis in LLC-$PK_1$ cells increases calcitonin-induced plasminogen activator gene transcription and mRNA stability. *Biochem. J.*, **242**, 387–392.

ALTUS, M.S. & NAGAMINE, Y. (1991). Protein synthesis inhibition stabilizes urokinase-type plasminogen activator mRNA. *J. Biol. Chem.*, **266**, 21190–21196.

BLASI, F., VASSALLI, J.D. & DANO, K. (1987). Urokinase-type plasminogen activator: proenzyme, receptor, and inhibitors. *J. Cell Biol.*, **104**, 801–804.

BUSSO, N., BELIN, D., FAILLY-CREPIN, C. & VASSALLI, J.D. (1986). Plasminogen activators and their inhibitors in a human mammary cell line (HBL-100). Modulation by glucocorticoids. *J. Biol. Chem.*, **261**, 9303–9315.

BUSSO, N., COLLART, M., VASSALLI, J.D. & BELIN, D. (1987). Antagonist effect of RU486 on transcription of glucocorticoid-regulated genes. *Exp. Cell Res.*, **173**, 425–430.

DANO, K., ANDREASEN, P.A., GRONDAHL-HANSEN, J., KRISTENSEN, P., NIELSEN, L.S. & SKRIVER, L. (1985). Plasminogen activators, tissue degradation, and cancer. *Adv. Cancer Res.*, **44**, 139–266.

HENDERSON, B.R. & KEFFORD, R.F. (1991). Conservation and potential role of RNA-instability motifs in urokinase gene 3'-untranslated sequences. *J. Natl Cancer Inst.*, **83**, 1103–1104.

HENDERSON, B.R., MCDONALD, D.A. & KEFFORD, R.F. (1992*a*). Post-transcriptional regulation of urokinase plasminogen activator gene expression occurs in the nucleus of BC1 rat mammary tumor cells. *Int. J. Cancer*, **50**, 918–923.

HENDERSON, B.R., TANSEY, W.P., PHILLIPS, S.M., RAMSHAW, I.A. & KEFFORD, R.F. (1992*b*). Transcriptional and posttranscriptional activation of urokinase plasminogen activator gene expression in metastatic tumor cells. *Cancer Res.*, **52**, 2489–2496.

HENTZE, M.W. (1991). Determinants and regulation of cytoplasmic mRNA stability in eukaryotic cells. *Biochim. Biophys. Acta*, **1090**, 281–292.

JONAT, C., RAHMSDORF, H.J., PARK, K.K., CATO, A.C., GEBEL, S., PONTA, H. & HERRLICH, P. (1990). Antitumor promotion and antiinflammation: down modulation of AP-1 (Fos-Jun) activity by glucocorticoid hormone. *Cell*, **62**, 1189–1204.

LEE, S.W., TSOU, A.P., CHAN, H., THOMAS, J., PETRIE, K., EUGUI, E.M. & ALLISON, A.C. (1988). Glucocorticoids selectively inhibit the transcription of the interleukin 1$\beta$ gene and decrease the stability of interleukin 1$\beta$ mRNA. *Proc. Natl Acad. Sci. USA*, **85**, 1204–1208.

MARODER, M., MARTINOTTI, S., VACCA, A., SCREPANTI, I., PETRANGELI, E., FRATI, L. & GULINO, A. (1990). Post-transcriptional control of c-*myc* proto-oncogene expression by glucocorticoid hormones in human T lymphoblastic leukemic cells. *Nucleic Acids Res.*, **18**, 1153–1157.

MEDCALF, R.L., VAN DEN BERG, E. & SCHLEUNING, W.-D. (1988). Glucocorticoid modulated gene expression of tissue- and urinary-type plasminogen activator and plasminogen activator inhibitor 1 and 2. *J. Cell Biol.*, **106**, 971–978.

MUKAIDA, N., ZACHARIAE, C.C.O., GUSELLA, G.L. MATSUSHIMA, K. (1991). Dexamethasone inhibits the induction of monocyte chemotactic-activating factor production by IL-1 or tumor necrosis factor. *J. Immunol.*, **146**, 1212–1215.

PEPPEL, K., VINCI, J.M. & BAGLIONI, C. (1991). The AU-rich sequences in the 3' untranslated region mediate the increased turnover of interferon mRNA induced by glucocorticoids. *J. Exp. Med.*, **173**, 349–355.

RORTH, P., NERVLOV, C., BLASI, F. & JOHNSEN, M. (1990). Transcription factor PEA3 participates in the induction of urokinase plasminogen activator transcription in murine keratinocytes stimulated with epidermal growth factor or phorbol ester. *Nucleic Acids Res.*, **18**, 5009–5017.

SCHULER, G.D. & COLE, M.D. (1988). GM-CSF and oncogene mRNA stabilities are independently regulated *in trans* in a mouse monocytic tumor. *Cell*, **55**, 1115–1122.

SHAW, G. & KAMEN, R. (1986). A conserved AU sequence from the 3' untranslated region of GM-CSF mRNA mediates selective mRNA degradation. *Cell*, **46**, 659–667.

ZIEGLER, A., HAGMAN, J., KIEFER, B. & NAGAMINE, Y. (1990). $Ca^{2+}$ potentiates cAMP-dependent expression of urokinase-type plasminogen activator gene through a calmodulin-and protein kinase C-independent mechanism. *J. Biol. Chem.*, **265**, 21194–21201.